# Prognostic Implications of Chronic Kidney Disease Stage on Outcomes After Percutaneous Coronary Intervention

**DOI:** 10.3390/jcdd13010004

**Published:** 2025-12-20

**Authors:** Keren Skalsky, Yeela Talmor-Barkan, Edward Itelman, Tsahi T. Lerman, Assaf Rotmensh, Leor Perl, Alon Shechter, Yaron Shapira, Arthur Shiyovich, Ran Kornowski, Amos Levi

**Affiliations:** 1 Department of Cardiology, Rabin Medical Center, Petach-Tikva 4941492, Israel; 2 Gray Faculty of Medical and Health Sciences, Tel Aviv University, Tel Aviv 02115, Israel; 3 Division of Cardiovascular Medicine, Department of Medicine, Brigham and Women’s Hospital, Harvard Medical School, Boston, MA 02115, USA; 4 Department of Radiology, Brigham and Women’s Hospital, Harvard Medical School, Boston, MA 02115, USA

**Keywords:** percutaneous coronary intervention, chronic kidney disease, myocardial infarction, target vessel revascularization

## Abstract

Aims: Chronic kidney disease (CKD) is associated with adverse cardiovascular outcomes, yet few contemporary studies stratify outcomes by specific CKD stages in the era of modern percutaneous coronary intervention (PCI) techniques and new-generation drug-eluting stents (DESs). We aim to assess the relationship between CKD and post-PCI outcomes in an updated, stage-specific, and long-term cohort. Methods: We retrospectively analyzed 11,489 patients who underwent PCI between 2010 and 2020. Kidney function was classified as preserved (eGFR ≥ 60 mL/min/1.73 m^2^), stage III CKD (eGFR 30–59), or stage IV/V CKD (eGFR < 30) using the CKD-EPI equation. The primary endpoint was a composite of all-cause mortality, non-fatal myocardial infarction (MI), and target vessel revascularization (TVR) at 1 year; secondary endpoints included individual components and outcomes through 5 years. Associations were evaluated using multivariable Cox regression. Results: Stage III and stage IV/V CKD were present in 18% and 5.6% of patients, respectively. At 1 year, both stage III (HR 2.13, *p* < 0.01) and stage IV/V CKD (HR 4.91, *p* < 0.01) were associated with higher risk of the composite endpoint. Mortality rose sharply with CKD severity (33% in stage IV/V vs. 4% in preserved renal function), and MI risk was significantly higher in stage IV/V CKD. These associations persisted after 5 years. Unadjusted TVR risk was higher in stage IV/V CKD but lost significance after adjustment. Conclusions: CKD, particularly in advanced stages, is independently associated with increased mortality and MI after PCI, with effects persisting in the long term. While advanced CKD showed higher unadjusted TVR risk, this was not independent after adjustment. These findings support individualized treatment strategies and extended follow-up in PCI patients with CKD.

## 1. Introduction

Chronic kidney disease (CKD) is a well-established independent risk factor for adverse cardiovascular outcomes, including coronary artery disease (CAD) [1,2]. The prevalence and severity of CAD increase progressively as kidney function declines, with patients in advanced stages of CKD (stages IV/V) facing particularly high risks of cardiovascular events and mortality compared to those with preserved renal function [3,4]. Despite advances in percutaneous coronary intervention (PCI) techniques, current evidence suggests that individuals with CKD, especially those with advanced disease, remain at an elevated risk of complications such as target vessel revascularization (TVR) and myocardial infarction (MI) following complex PCI [5,6]. While the association between CKD and adverse cardiovascular outcomes is well established, fewer contemporary studies stratify outcomes by specific CKD stages in the era of modern PCI techniques and second-/third-generation DES.

Understanding how varying degrees of renal dysfunction affect post-PCI outcomes is essential for optimizing management strategies in this high-risk population. In this study, we aimed to evaluate the incidence of all-cause mortality, TVR and MI following PCI across different stages of CKD.

## 2. Materials and Methods

### 2.1. Study Population

We conducted a retrospective analysis of 11,489 patients who underwent PCI between 2010 and 2020 at Rabin Medical Center. CKD was defined using the CKD-EPI equation, categorizing patients into preserved kidney function (eGFR ≥ 60 mL/min/1.73 m^2^, including CKD stage I/II), CKD stage III (eGFR 30–59 mL/min/1.73 m^2^) and CKD stage IV/V (eGFR < 30 mL/min/1.73 m^2^), also referred as advanced CKD [7]. The study included all PCI with exception, including patients with recurrent lesions and post CABG.

### 2.2. Follow-Up and Outcomes

Patients were followed from the time of PCI until death or up to five years, whichever occurred first. The primary endpoint was a composite of all-cause mortality, non-fatal MI and TVR at one year. Secondary outcomes included each of the components of the primary outcome in separate. Long-term outcomes up to five years were assessed. Mortality data were obtained from the Israeli Ministry of the Interior’s Population Registry.

### 2.3. Data Collection and Definitions

CKD diagnosis was based on the most recent creatinine level recorded within three months prior to the PCI procedure. eGFR was calculated using the CKD-EPI equation [8]. Patients with an eGFR of 30–59 mL/min/1.73 m^2^ were classified as CKD stage III, those with 15–29 mL/min/1.73 m^2^ as stage IV, and those with eGFR < 15 mL/min/1.73 m^2^ or requiring renal replacement therapy as stage V [7].

Baseline comorbidities were recorded according to physician documentation at the time of PCI. Obstructive coronary artery disease was defined as angiographic stenosis ≥ 70% in a vessel with a diameter ≥ 2 mm, ≥50% in the left main, or a fractional flow reserve (FFR) < 0.80. Severe left ventricular dysfunction was defined as an ejection fraction < 30% based on the initial echocardiogram during hospitalization [9]. Reinterventions were captured as TVR, defined as repeat PCI or CABG of the index vessel.

### 2.4. Statistical Analysis

Continuous variables were presented as means ± standard deviation (SD) or medians with interquartile ranges (IQR), as appropriate. Group comparisons for continuous variables were performed using one-way ANOVA or the Kruskal–Wallis test, depending on data distribution. Categorical variables were reported as counts and percentages and compared using the chi-squared test. For pairwise comparisons between two groups, the chi-squared test was used for categorical variables, and either Student’s *t*-test or the Mann–Whitney–Wilcoxon test was applied for continuous variables, based on the normality of the data’s distribution. As a secondary analysis, we performed a competing risk analysis to account for the influence of death on the incidence of TVR and re-MI. The Fine and Gray sub distribution hazard model was used to estimate the cumulative incidence function, treating death as a competing event. Sub distribution hazard ratios (sHRs) and 95% confidence intervals (CIs) were calculated.

All statistical tests were two-sided, and a *p*-value < 0.05 was considered significant. Hazard ratios (HR) are presented with 95% confidence intervals (CI). Kaplan–Meier survival curves were generated, and the log-rank test was used to compare survival rates across groups at one and five years. Cox proportional hazards models were used to assess the association between CKD stage and outcomes (TVR, MI, mortality), adjusting for relevant covariates identified in univariate analyses (*p* < 0.05). Final adjusted models included age, sex, diabetes, prior stroke, COPD, PVD, ACS/MI at presentation, radial vs. femoral access, LVEF, atrial fibrillation, and congestive heart failure. All analyses were performed using R (version 4.0.0, RStudio, Vienna, Austria).

## 3. Results

### 3.1. Study Population and Strata

Between 2010 and 2020, 11,489 patients underwent PCI at Rabin Medical Center. Among these, 2708 (23.6%) were identified as having CKD, including 2063 patients (18%) with stage III CKD and 645 patients (5.6%) with stage IV/V CKD.

The study population was predominantly older adults. The mean age was 72.5 years for patients with CKD stage IV/V, 75.0 years for stage III, and 63.4 years for those with preserved renal function. Approximately one-third of the cohort was female, with lower proportions in earlier CKD stages. Cardiovascular risk factors, including diabetes mellitus, hypertension, smoking, and chronic obstructive pulmonary disease (COPD) were more prevalent with increasing CKD severity. Likewise, prior stroke and peripheral vascular disease were more common in advanced CKD stages (Table 1).

Cardiac conditions such as congestive heart failure and reduced ejection fraction were also more frequent in patients with stage IV/V CKD. Additionally, lipid profiles (total cholesterol and LDL) tended to be lower among patients with advanced CKD.

PCI indications and procedural characteristics differed by CKD stage. The proportion of interventions for acute coronary syndromes decreased from 67.9% in patients with preserved renal function to 55.5% in those with stage IV/V CKD. Left main coronary interventions were more frequent in advanced CKD, while intervention distribution across other coronary territories was similar. Femoral artery access was used more often than radial access in patients with advanced CKD (Table 1).

Drug-eluting stents were used almost exclusively. Several drug-eluting stent platforms were employed during the study period, including Xience (everolimus-eluting stent, Abbott Vascular, Santa Clara, CA, USA), Onyx (zotarolimus-eluting stent, Medtronic, Santa Rosa, CA, USA), BioMatrix (biolimus-A9–eluting stent, Biosensors International, Singapore), Orsiro (sirolimus-eluting stent, Biotronik, Bülach, Switzerland), and Ultimaster (sirolimus-eluting stent, Terumo, Tokyo, Japan). Xience was the most commonly used platform.

### 3.2. Follow-Up and Outcomes

**Figure 1:**
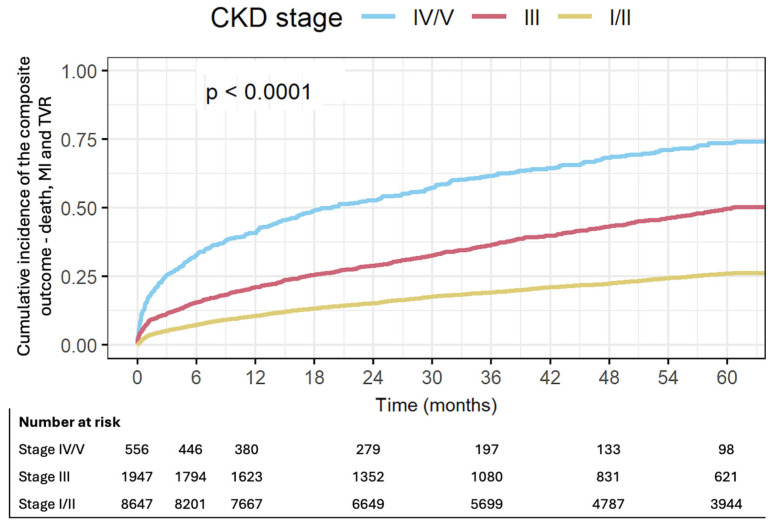
Cumulative incidence of the composite outcome—death, MI and TVR by CKD stage.

CKD stages III and IV/V were associated with increased rates of the composite outcome including all-cause mortality, non-fatal MI and TVR at 1 year. Patients with CKD stage III were at 2.13 fold risk for mortality, MI or TVR (95% CI 1.9–2.4, *p* < 0.01). This risk increased to 4.91 among patients with CKD stage IV/V (95% CI 4.28–5.63, *p* < 0.01) (Table 2, Figure 1).

**Figure 2:**
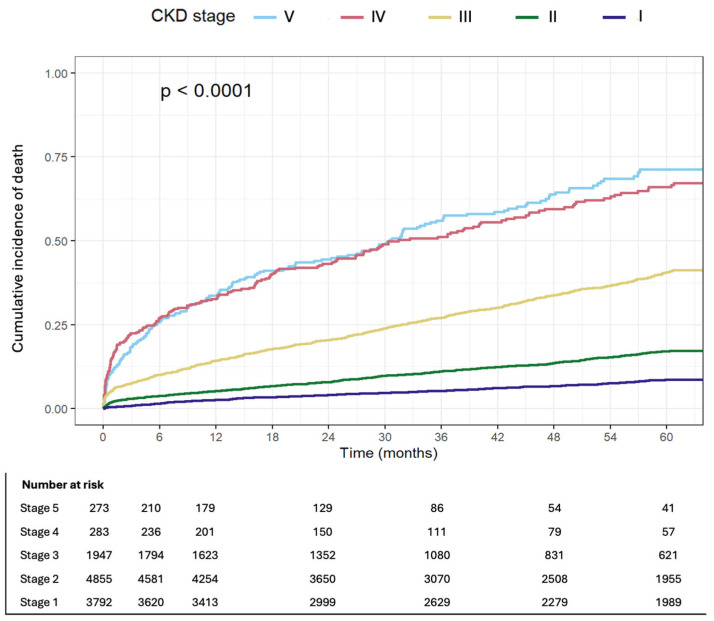
Cumulative incidence of death by CKD stage.

All-cause 33. in patients with CKD stage IV/V, compared to 14.1% in patients with CKD stage III and 4% among patients with preserved kidney function. Kaplan–Meier curves depicting mortality by CKD stage are presented in Figure 2.

The increased rates of non-fatal MI were consistent in all CKD stages, with HR of 2.29 (95% CI 1.78–2.95, *p* < 0.01) at stage IV/V and 1.31 (95% CI 1.1–1.6, *p* < 0.01) at stage III CKD. CKD stage IV/V was associated with an increased risk of TVR (HR 1.67, 95% CI 1.07–2.61, *p* = 0.02), whereas this association was not significant for CKD stage III.

The associations of CKD with the composite primary outcome, as well as all-cause mortality and non-fatal MI continued through the five-year follow-up. Patients with advanced CKD had 4.63 fold risk of experiencing death, MI or TVR (95% CI 4.16–5.15) compared to patients with preserved kidney function. This risk was 2.22 fold higher for patients with CKD stage III (95% CI 2.05–2.4).

Five-year mortality was 57% in patients with CKD stage IV/V, compared to 10.5% in patients with preserved kidney function. The hazard ratio for MI at five years was 2.09 (95% CI 1.7–2.57, *p* < 0.001) and 1.29 (95% CI 1.2–1.47, *p* < 0.001) in advanced CKD and at CKD stage III, respectively.

At five years, the risk of TVR in CKD stage IV/V did not reach statistical significance (HR 1.37, 95% CI 0.94–2.02, *p* = 0.104) (Table 2).

### 3.3. Multivariable and Competing Risk Analysis

After adjustment, stage IV/V remained independently associated with the primary composite outcome at 1 year (HR 3.00, 95% CI 2.58–3.49, *p* < 0.001) and 5 years (HR 2.78, 95% CI 2.48–3.13, *p* < 0.001). The adjusted risk for CKD stage III was 1.46 (95% CI 1.29–1.65, *p* < 0.001) at 1 year and 1.49 (95% CI 1.37–1.63) at 5 years (Table 3).

**Table 4:** Multivariable analysis for all-cause mortality at 1 year and at 5 years.

Variable	1-Year HR (95% CI)	*p*-Value	5-Year HR (95% CI)	*p*-Value
CKD Stage				
IV/V	4.73 (3.90–5.73)	<0.001	4.13 (3.60–4.73)	<0.001
III	2.18 (1.85–2.58)	<0.001	2.15 (1.93–2.38)	<0.001
Adjusted Variables				
Age (per year)	1.03 (1.02–1.03)	<0.001	1.03 (1.03–1.03)	<0.001
Female gender	1.29 (1.11–1.49)	<0.001	1.20 (1.09–1.32)	<0.001
Diabetes mellitus	1.01 (0.88–1.16)	0.913	1.20 (1.10–1.32)	<0.001
MI or ACS (non-elective PCI)	1.17 (1.01–1.35)	0.032	0.97 (0.89–1.07)	0.536
Moderate-to-severe LV dysfunction	2.46 (2.13–2.84)	<0.001	2.09 (1.90–2.30)	<0.001
Atrial fibrillation	0.58 (0.42–0.80)	<0.001	0.94 (0.80–1.10)	0.422
Peripheral vascular disease	1.49 (1.17–1.90)	0.001	1.78 (1.52–2.09)	<0.001
COPD	1.57 (1.31–1.88)	<0.001	1.64 (1.45–1.85)	<0.001
Radial approach	0.50 (0.44–0.58)	<0.001	0.68 (0.62–0.75)	<0.001
Prior stroke	1.17 (0.95–1.44)	0.145	1.29 (1.13–1.48)	<0.001
Congestive heart failure	0.65 (0.51–0.83)	<0.001	1.06 (0.94–1.21)	0.355

The increased risk of MI in CKD stage IV/V remained significant after multivariable adjustment at both 1 and 5 years. However, CKD stage III was not independently associated with increased MI risk (Table 5).

The association between advanced CKD and TVR was attenuated after adjustment, with no independent association observed for either 1-year or 5-year follow-up (Table 6).

Other predictors of increased mortality included older age (≥65 years), female sex, peripheral vascular disease, diabetes, COPD, femoral vs. radial access, and severe left ventricular dysfunction. These findings were consistent in competing risk models (Supplementary Materials Table S1).

## 4. Discussion

In this large retrospective cohort study, we evaluated the impact of CKD severity on cardiovascular outcomes following PCI. Worsening CKD stage was strongly and consistently associated with adverse outcomes. Advanced CKD (stage IV/V) conferred more than a fourfold higher risk of the composite endpoint of death, MI, or TVR at both 1 and 5 years, while stage III CKD carried approximately a twofold increased risk. Mortality risk rose steeply with declining renal function, reaching 57% at 5 years in stage IV/V. Although MI risk remained elevated in advanced CKD after adjustment, the association with TVR was attenuated and no longer significant.

These results are strongly supported by the literature. Multiple large cohort studies and registries have demonstrated a stepwise increase in mortality and major adverse cardiovascular events (MACE) with worsening CKD stage after PCI, even after multivariable adjustment for confounders [10,11,12,13]. For example, the J-MINUET study found that three-year mortality and MACE rates rise sharply from patients with preserved kidney function to those with moderate and severe CKD, and that CKD stage improved risk prediction models for adverse outcomes [12]. Those findings are also supported by meta-analysis demonstrating that CKD is an independent predictor of mortality and MI after PCI, with risk increasing as eGFR declines [14].

Several pathophysiological mechanisms probably contribute to the poor post-PCI outcomes observed in patients with CKD. Chronic kidney dysfunction is associated with systemic inflammation, oxidative stress, endothelial dysfunction, and as a result accelerated atherosclerosis, increased risk for restenosis or therefore recurrent ischemia [15,16,17]. Additional mechanism is uremia-related platelet dysfunction and impaired drug metabolism that may compromise both procedural success and antiplatelet efficacy [18,19].

The increased rate of femoral access and higher interventional complexity in patients with advanced CKD stages, as seen in our cohort, may further contribute to the increased procedural risk and worse outcomes.

The association between CKD and restenosis or stent thrombosis remains incompletely understood. Much of the existing evidence is based on earlier-generation stent platforms and outdated interventional techniques. The SYNTAX trial, which utilized paclitaxel-eluting stents, reported worse outcomes and higher restenosis rates in patients with eGFR < 30 mL/min/1.73 m^2^ or dialysis dependence. However, its five-year follow-up failed to establish a significant link between CKD and TVR [20]. Other trials, including a 2016 Korean study, showed that individuals with advanced CKD experienced higher rates of target lesion failure, even with second-generation drug-eluting stents [5]. In contrast, findings from the HORIZONS-AMI trial indicated no significant association between CKD and TVR at 30 days, 1 year, or 3 years [14,21]. Furthermore, in that same trial, no difference in TVR rates was observed between bare-metal and drug-eluting stents in CKD patients at 3-year follow-up [21]. In our cohort, the association between CKD and TVR was modest. A statistically significant association was observed at one year in patients with stage IV/V CKD, but this association diminished over time and was no longer significant after adjusting for confounding variables and competing risks. Multivariate analysis revealed that neither stage III nor stage IV/V CKD independently predicted TVR. These findings suggest that MI in this cohort was more likely driven by de novo lesions rather than stent failure. Advanced CKD may serve as a surrogate marker for higher TVR risk, rather than being a direct causal factor.

In the present cohort, the association between CKD and TVR was modest. A statistically significant association was observed at one year among patients with stage IV/V CKD; however, this association attenuated over time and was no longer significant after adjustment for confounding variables and competing risks. On multivariable analysis, neither stage III nor stage IV/V CKD independently predicted TVR. These findings suggest that subsequent myocardial infarction in this population may be more frequently attributable to de novo coronary lesions rather than stent-related failure, consistent with the systemic inflammatory milieu and accelerated atherosclerotic progression observed in advanced CKD. Additional explanations may include differences in lesion complexity, as reflected by higher rates of left main and graft PCI in advanced CKD, as well as lower radial access use and a greater comorbidity burden, which may influence the threshold for repeat revascularization.

Importantly, although competing risk methodology was applied, the substantially higher mortality observed in patients with advanced CKD continues to exert a strong influence on long-term revascularization outcomes. This observation supports the interpretation that advanced CKD may function as a marker of overall disease severity and adverse vascular biology rather than serving as an independent causal determinant of TVR.

Our study has several notable strengths that enhance the robustness and relevance of these findings. First, it represents one of the largest and most contemporary real-world cohorts to date evaluating the prognostic impact of CKD stage on PCI outcomes, encompassing over 11,000 patients treated within the last decade. Second, the analysis specifically examined TVR as a distinct endpoint, which has been underreported in prior CKD-PCI research. Importantly, the study reflects current clinical practice in the era of latest-generation drug-eluting stents, advanced interventional techniques, and contemporary pharmacological therapies, thereby providing relevant insights for present-day patient management. The inclusion of both short- and long-term follow-up strengthens the validity of the findings and allows for a comprehensive assessment of temporal trends in risk. Finally, the use of detailed multivariable and competing risk analyses enhances the robustness of the results and supports their applicability to a broad PCI population.

As CKD is becoming a growing global health burden, a substantial proportion of patients undergoing PCI have some degree of renal impairment. Renal dysfunction is independently associated with increased cardio-vascular risk. Whether PCI modifies this risk remains uncertain.

The ISCHEMIA-CKD trial evaluated elective patients with advanced CKD (stages 4–5 or dialysis-dependent) and failed to find benefit of invasive strategy of revascularization (PCI or CABG) over optimal medical therapy. However, interpretation of these results should be cautious, as the trial excluded patients with severe symptoms, left main disease, or systolic heart failure. Additionally, crossover between treatment arms was frequent, and peri-procedural MI rates were higher in the intervention group compared to the conservative treatment group [22,23].

Unlike elective procedures, in the acute setting, PCI remains a necessary and often life-saving intervention unrelated to CKD severity. Data from the SWEDEHEART registry demonstrated improved one-year survival with an early invasive strategy in patients with NSTEMI and CKD stages 2–4; nevertheless, the benefit of invasive strategy declined with progression of the kidney failure and was less evident in stage 5 CKD and in dialysis patients [24]. However, more recent and up to date study have supported invasive management in patients with acute MI in all stages of CKD patients including those on hemodialysis, showing reduced in-hospital mortality [25]. The present study was not designed to compare PCI with conservative management in patients with CKD. Rather, it describes clinical outcomes among CKD patients who underwent PCI. Accordingly, our findings should not be interpreted as suggesting harm from PCI, but instead indicate that CKD remains associated with residual adverse risk despite revascularization. Although long-term mortality was high among patients with stage IV/V CKD, current evidence does not support withholding PCI in the setting of acute myocardial infarction on the basis of renal dysfunction alone. In elective settings, our results underscore the importance of careful patient selection and multidisciplinary decision-making.

Based on the study results and previous publications, we support a risk-adjusted approach in managing patients with CKD undergoing PCI, taking into consideration elective vs. emergent intervention, access site and the severity of renal impairment. In elective cases, strategies may include intensified surveillance with functional testing or symptom-triggered imaging, optimization of medical therapy, consideration of access site, and possibly earlier involvement of nephrology and multidisciplinary heart-kidney teams. Procedural decisions should balance benefits against long-term survival projections, informed by evidence from the ISCHEMIA-CKD trial, while acknowledging its limitations, and the observed TVR rates from this analysis and earlier studies. In acute presentations, clinicians should refrain from postponing PCI out of concern for potential renal injury, a practice sometimes referred to as “renalism” [26].

Several limitations merit consideration. First, this was a retrospective, observational, single-center study. Although the cohort was large and analyses incorporated multivariable adjustment and competing risk methodology, residual confounding cannot be fully excluded. Second, CKD classification was based on estimated glomerular filtration rate measured prior to PCI and did not include albuminuria or proteinuria, which may have resulted in misclassification or underestimation of renal disease severity. Third, data on medication adherence, contrast volume, nephroprotective strategies and contrast-associated kidney injury or CKD progression were unavailable, and these factors may have influenced clinical outcomes. In addition, the study did not include a comparator group of patients with CKD managed without PCI, precluding assessment of the relative benefits or risks of revascularization versus conservative treatment in this population. Finally, although follow-up was comprehensive, cause-specific mortality data were not available, limiting our ability to distinguish cardiovascular from non-cardiovascular causes of death.

## 5. Conclusions

CKD, particularly in advanced stages, is independently associated with increased mortality and MI after PCI, with effects persisting long-term. While advanced CKD showed higher unadjusted TVR risk, this was not independent after adjustment. These findings support individualized treatment strategies and extended follow-up in PCI patients with CKD.

## Figures and Tables

**Table 1:** Baseline demographics, clinical history, and procedural characteristics of patients, stratified by CKD stage.

	Preserved Kidney Function (*n* = 8781)	Stage III (*n* = 2063)	Stage IV/V (*n* = 645)	*p*
Demographics				
age (years)	63.4 (11.6)	75 (10.1)	72.5 (12.9)	0.000
Female gender (%)	20.1	33.7	33	0.000
Cardiovascular risk factors				
DM (%)	40.8	54.9	70.7	0.000
HTN (%)	67.2	87.6	94.4	0.000
Smoking History (%)	38.6	22	19.2	0.000
COPD (%)	7.6	11.8	14	0.000
PVD (%)	4.1	7.4	15.5	0.000
Prior Stroke (%)	5.6	10.4	14.6	0.000
Cardiac diseases				
Known CHF (%)	7.7	14.9	11.6	0.000
EF (%)	54.9 (8.9)	52.7 (10.3)	49.9 (11.5)	0.000
Known Atrial Fibrillation (%)	4.4	7.6	8.2	0.000
S/P CABG (%)	9.1	15.7	18.8	0.000
Other diseases				
Prior Malignancy (%)	9.7	18.2	22.2	0.000
Active Malignancy (%)	4	5.5	2.9	0.002
Dementia (%)	1.5	4.7	5.1	0.000
Blood tests results				
Total Cholesterol (mg/dL)	170.8 (44.7)	156.9 (44.2)	147.8 (44.3)	0.000
HDL (mg/dL)	41.5 (12.4)	42.8 (13.9)	39.3 (12.8)	0.000
LDL (mg/dL)	99.7 (37.8)	85.7 (36.5)	78.2 (35.7)	0.000
Triglycerides (mg/dL)	153.7 (106.6)	143.1 (85.2)	157.6 (96.2)	0.001
HbA1C (%)	6.8 (1.7)	7.3 (1.8)	7.2 (1.9)	0.000
Hgb (g/dL)	13.7 (1.7)	12.4 (2)	10.7 (1.7)	0.000
MPV (fL)	8.9 (1.2)	9.1 (1.3)	9.2 (1.3)	0.000
Plt (10^3^/µL)	232.2 (73.7)	227.5 (86.1)	221.8 (84.9)	0.000
Creatinine (mg/dL)	0.9 (0.2)	1.4 (0.3)	4.3 (2.6)	0.000
Uric Acid (mg/dL)	5.8 (1.5)	7.2 (1.9)	7.7 (2.7)	0.000
WBC (10^3^/µL)	8.7 (4.7)	9.2 (9.4)	8.6 (4.1)	0.002
Coronary intervention				
Acute MI (%)	44.8	42.7	38	0.002
STEMI (%)	21.4	16.5	12.9	0.000
ACS (excluding MI) (%)	24	21.7	18.3	0.001
CX_treated (%)	30.6	30.8	34.1	0.183
LAD_treated (%)	48.3	46.2	42.8	0.009
RCA_treated (%)	32.4	28.1	29.1	0.000
LM_treated (%)	5.3	10.5	12.6	0.000
Graft treated (%)	2.1	5.6	5.7	0.000
Multivessel PCI (%)	10.3	10.2	12.2	0.308
CTO treated (%)	13.6	11.9	10.4	0.012
DES (%)	76.9	72.5	73	0.000
DEB (%)	2.9	3.3	3.3	0.494
Radial approach (%)	64.4	51.2	40.9	0.000

Abbreviations: DM—Diabetes Mellitus; HTN—Hypertension; COPD—Chronic Obstructive Pulmonary Disease; PVD—Peripheral Vascular Disease; CHF—Congestive Heart Failure; EF—Ejection Fraction; S/P CABG—Status Post Coronary Artery Bypass Grafting; HDL—High-Density Lipoprotein; LDL—Low-Density Lipoprotein; HgA1C—Hemoglobin A1C; Hgb—Hemoglobin; MPV—Mean Platelet Volume; Plt—Platelets; WBC—White Blood Cells; MI—Myocardial Infarction; STEMI—ST-Elevation Myocardial Infarction; ACS—Acute Coronary Syndrome; CX—Circumflex Artery; LAD—Left Anterior Descending Artery; RCA—Right Coronary Artery; LM—Left Main Artery; Graft—Coronary Artery Bypass Graft; PCI—Percutaneous Coronary Intervention; CTO—Chronic Total Occlusion; DES—Drug-Eluting Stent; DEB—Drug-Eluting Balloon.

**Table 2:** All-cause mortality, acute MI and TVR at 1 year and at 5 years in each CKD stage.

Outcomes	Preserved Kidney Function (*n* = 8781), %	Stage III (*n* = 2063), %	Stage IV/V (*n* = 645), %	*p*
Non-fatal MI, TVR and all-cause mortality at 1 year	10.3	20.6	40.8	0.000
All-cause mortality at 1 year	4	14.1	33	0.000
MI at 1 year	5.8	7.1	10.5	0.000
TVR at 1 year	2.4	2.3	3.3	0.387
Non-fatal MI and all-cause mortality at 1 year	9.4	20.1	40.2	0.000
All-cause mortality at 5 years	10.5	33.3	57.2	0.000
MI at 5 years	11.1	12.6	15.2	0.002
TVR at 5 years	4.7	4.8	4.3	0.901
Non-fatal MI and all-cause mortality at 5 years	20.1	40.8	63.3	0.000

Abbreviations: MI—Myocardial Infarction; TVR—Target Vessel Revascularization.

**Table 3:** Multivariable analysis for the composite outcome—all-cause mortality, MI and TVR at 1 year and at 5 years.

Variable	1-Year HR (95% CI)	*p*-Value	5-Year HR (95% CI)	*p*-Value
CKD Stage				
IV/V	3.00 (2.58–3.49)	<0.001	2.78 (2.48–3.13)	<0.001
III	1.46 (1.29–1.65)	<0.001	1.49 (1.37–1.63)	<0.001
Adjusted Variables				
Age (per year)	1.018 (1.014–1.022)	<0.001	1.020 (1.017–1.022)	<0.001
Female gender	1.15 (1.03–1.28)	0.015	1.07 (0.99–1.16)	0.107
Diabetes mellitus	1.14 (1.03–1.27)	0.010	1.27 (1.18–1.37)	<0.001
MI or ACS (non-elective PCI)	1.43 (1.29–1.60)	<0.001	1.17 (1.09–1.26)	<0.001
Moderate-to-severe LV dysfunction	1.76 (1.57–1.96)	<0.001	1.64 (1.52–1.78)	<0.001
Atrial fibrillation	0.92 (0.76–1.13)	0.432	1.08 (0.95–1.23)	0.218
Peripheral vascular disease	1.33 (1.09–1.63)	0.005	1.56 (1.35–1.79)	<0.001
COPD	1.28 (1.10–1.48)	0.001	1.36 (1.23–1.51)	<0.001
Radial approach	0.64 (0.58–0.70)	<0.001	0.76 (0.71–0.82)	<0.001
Prior stroke	1.23 (1.05–1.44)	0.012	1.25 (1.12–1.40)	<0.001
Congestive heart failure	1.13 (0.98–1.31)	0.101	1.32 (1.20–1.46)	<0.001

Abbreviations: ACS—Acute coronary syndrome; CKD—Chronic kidney disease; CI—Confidence interval; COPD—Chronic obstructive pulmonary disease; HR—Hazard ratio; LV—Left ventricular; MI—Myocardial infarction; PCI—Percutaneous coronary intervention; TVR—Target vessel revascularization All-cause mortality remained significantly elevated across CKD stages. In CKD stage IV/V, mortality risk was 4.73-fold higher at 1 year (95% CI 3.90–5.73, *p* < 0.001) and 4.13-fold higher at 5 years (95% CI 3.60–4.73, *p* < 0.001). For CKD stage III, the corresponding HRs were 2.18 (95% CI 1.85–2.58, *p* < 0.001) at 1 year and 2.15 (95% CI 1.93–2.38, *p* < 0.001) at 5 years (Table 4).

**Table 5:** Multivariable analysis for MI at 1 year and at 5 years.

Variable	MI at 1 Year HR (95% CI)	*p*-Value	MI at 5 Years HR (95% CI)	*p*-Value
CKD Stage				
IV/V	1.83 (1.40–2.39)	<0.001	1.68 (1.35–2.09)	<0.001
III	1.06 (0.87–1.29)	0.578	1.08 (0.93–1.26)	0.292
Adjusted Variables				
Age (per year)	1.01 (1.00–1.01)	0.147	1.00 (0.99–1.00)	0.894
Female gender	1.02 (0.86–1.21)	0.828	0.95 (0.83–1.08)	0.407
Diabetes mellitus	1.31 (1.13–1.53)	<0.001	1.42 (1.27–1.59)	<0.001
MI or ACS (non-elective PCI)	2.02 (1.69–2.42)	<0.001	1.65 (1.46–1.87)	<0.001
Moderate-to-severe LV dysfunction	1.09 (0.91–1.32)	0.350	1.13 (0.99–1.30)	0.076
Atrial fibrillation	1.42 (1.11–1.83)	0.006	1.48 (1.23–1.77)	<0.001
Peripheral vascular disease	1.07 (0.75–1.52)	0.722	1.26 (0.98–1.63)	0.072
COPD	0.95 (0.73–1.23)	0.687	1.00 (0.83–1.21)	0.979
Radial approach	0.77 (0.66–0.89)	<0.001	0.81 (0.73–0.90)	<0.001
Prior stroke	1.35 (1.07–1.72)	0.013	1.25 (1.04–1.50)	0.019
Congestive heart failure	2.00 (1.65–2.42)	<0.001	2.04 (1.77–2.35)	<0.001

Abbreviations: ACS—Acute coronary syndrome; CKD—Chronic kidney disease; CI—Confidence interval; COPD—Chronic obstructive pulmonary disease; HR—Hazard ratio; LV—Left ventricular; MI—Myocardial infarction; PCI—Percutaneous coronary intervention.

**Table 6:** Multivariable analysis for TVR at 1 year and at 5 years.

Variable	1-Year HR (95% CI)	*p*-Value	5-Year HR (95% CI)	*p*-Value
CKD Stage				
IV/V	1.16 (0.73–1.85)	0.530	1.08 (0.73–1.60)	0.714
III	0.74 (0.53–1.04)	0.084	0.97 (0.76–1.23)	0.774
Adjusted Variables				
Age (per year)	1.01 (1.00–1.02)	0.077	1.00 (0.99–1.01)	0.894
Female gender	0.85 (0.64–1.13)	0.267	0.82 (0.66–1.02)	0.069
Diabetes mellitus	1.88 (1.47–2.41)	<0.001	1.77 (1.48–2.11)	<0.001
MI or ACS (non-elective PCI)	1.49 (1.15–1.95)	0.003	1.18 (0.98–1.42)	0.073
Moderate-to-severe LV dysfunction	1.22 (0.92–1.63)	0.169	1.06 (0.85–1.33)	0.589
Atrial fibrillation	1.14 (0.75–1.75)	0.534	1.40 (1.05–1.88)	0.022
Peripheral vascular disease	0.79 (0.42–1.49)	0.464	1.12 (0.74–1.69)	0.598
COPD	0.95 (0.64–1.43)	0.823	0.96 (0.71–1.30)	0.787
Radial approach	0.81 (0.63–1.02)	0.077	0.93 (0.78–1.10)	0.382
Prior stroke	1.58 (1.10–2.26)	0.012	1.44 (1.09–1.90)	0.010
Congestive heart failure	2.16 (1.60–2.91)	<0.001	1.96 (1.56–2.45)	<0.001

Abbreviations: ACS—Acute coronary syndrome; CKD—Chronic kidney disease; CI—Confidence interval; COPD—Chronic obstructive pulmonary disease; HR—Hazard ratio; LV—Left ventricular; MI—Myocardial infarction; PCI—Percutaneous coronary intervention; PVD—Peripheral vascular disease; TVR—Target vessel revascularization.

## Data Availability

The data presented in this study are available on request from the corresponding author.

## Supplementary Materials

The following supporting information can be downloaded at https://www.mdpi.com/article/10.3390/jcdd13010004/s1, Table S1: Fine and grey competing risk models.
