# Supplementary material for: Prognostic Implications of Chronic Kidney Disease Stage on Outcomes After Percutaneous Coronary Intervention
Source: J Cardiovasc Dev Dis. 2025 Dec 20;13(1):4. doi: 10.3390/jcdd13010004 (PMC12842264; doi:10.3390/jcdd13010004)
Supplement: Supplementary file 1 [file jcdd-13-00004-s001.zip › jcdd-4026265-supplementary.pdf]

Supplementary Table S1: Fine and grey competing risk models

	MI at 1 year			MI at 5 years		
	**HR**	**95% CI**	**p-value**	**HR**	**95% CI**	**p-value**
**CKD Stage**						
CKD Stage 4/5	1.31	1.06-1.1.6	0.01	1.87	1.45-2.41	<0.001
CKD Stage 3	1.1	0.97-1.25	0.14	1.24	1.03-2.29	0.02
	**TVR at 1 year**			**TVR at 5 years**		
**CKD Stage**						
CKD Stage 4/5	0.88	0.61-1.27	0.5	1.35	0.86-2.11	0.19
CKD Stage 3	0.97	0.79-1.19	0.76	0.96	0.7-1.31	0.8
